# Impact of Environmental Thermal Stimulation on Activation of Hypothalamic Neuronal Nitric Oxide Synthase during the Prenatal Ontogenesis in Muscovy Ducks

**DOI:** 10.1100/2012/416936

**Published:** 2012-04-19

**Authors:** Valery Dunai, Barbara Tzschentke

**Affiliations:** ^1^Institute of Biology, Humboldt-University of Berlin, Philippstraße 13, 10115 Berlin, Germany; ^2^Department of Human Ecology, Belorussian State University, prospect Nezavisimosti 4, 220050, Minsk, Belarus

## Abstract

The aim of the study is to investigate the influence of prenatal temperature stimulation on neuronal NO synthase (nNOS) expression in the anterior hypothalamus of Muscovy duck embryos. Experiments were performed on embryonic day (E) E20, E23, E28, and E33 using histochemistry for identification of the nicotinamide adenine dinucleotide phosphate-diaphorase (NADPH-d) as marker of NOS-containing neurons. Until the experiments, all duck embryos were incubated under standard temperature conditions (37.5°C). During 3 hours before the start of the experiments, one group was incubated at 37.5°C (control group), the second was warm-experienced at 39°C, and the third was cold-experienced at 34°C. In normal and warm-incubated duck embryos, nNOS activity could be first detected on E23. Particularly, after cold stimulation, a significant increase in nNOS activity was found in all embryos investigated even on day 20. Warm stimulation obviously induces the opposite effect, but at later embryonic age (E33). It can be concluded that probably in late-term bird embryos NO acts as a mediator of the neuronal cold pathway in the anterior hypothalamus, which might be improved by prenatal cold stimulation.

## 1. Introduction

In the course of prenatal and early postnatal ontogeny, environmental factors may influence the development of the respective physiological control systems for the entire life period, especially by changes in neural organization and expression of related effector genes [1, 2]. During early ontogeny, nitric oxide (NO) plays a very important role in the formation of central nervous networks, for example, as a key player that mediated epigenetic mechanisms in developing neurons [3]. NO is involved in neurotransmitter release [4, 5] and synaptic plasticity throughout life, for example, [6–9]. NO is produced by activation of nitric oxide synthase (NOS), which converts L-arginine to L-citrulline and NO [10]. The marker for NOS-positive neurons is nicotinamide adenine dinucleotide phosphate-diaphorase (NADPH-d) [11].

In mammals and birds, during different developmental periods, NO plays a crucial role in regulation of various physiological functions, which are mediated by the hypothalamus, like thermoregulation [12–16], water balance [17, 18] and fever [19, 20], feeding behaviour [21–25], energy balance [26], and cardiovascular regulation [27–29]. However, in birds under thermoneutral conditions or heat load, for instance, the thermoregulatory effect of NO seems to be different from that in mammals [16], in which central NO mostly induces heat loss responses and finally decreases body temperature [30].

Investigations on localization of NADPH-d in the chicken brain show that the pattern of expression of NADPH-d, and thus of nNOS, within the avian and mammalian brain might be largely conserved [31]. In the adult chicken, NADPH-d-containing neurons were found in the lateral hypothalamus, dorsal hypothalamic area, hypothalamic periventricular nucleus, paraventricular nucleus, and mammillary area [32].

The present study is related to our project on the influence of the prenatal environment on the prenatal and postnatal development of body functions with the thermoregulatory system as an example and the precocial bird as a model. Because of its long incubation time of 35 days, the Muscovy duck embryo fits very good for such investigations. Further, in our research group the early development of neuronal and peripheral mechanisms of the thermoregulatory system as well as other body functions was intensively investigated in the Muscovy duck [33–37]. At the end of incubation in the Muscovy duck, hypothalamic neuronal thermoregulatory mechanisms are well developed [34]. Further, changes in the prenatal thermal environment may stimulate the development of central thermoregulation with long-term influence when applied during critical developmental periods (epigenetic temperature adaptation) [1, 35, 38, 39]. We assume a crucial role of NO in environmental-induced neuronal plasticity also in the birds' thermoregulatory network during different developmental periods. Related to this hypothesis, the aim of the actual study is to investigate the influence of prenatal temperature stimulation on nNOS expression in the preoptic area of the anterior hypothalamus (PO/AH) of Muscovy duck embryos during second half of incubation using histochemistry.

## 2. Material and Methods

### 2.1. Incubation of Duck Embryos

In the Muscovy duck the total incubation time comprises 35 days. Experiments were carried out in Muscovy ducks during second half of incubation on the embryonic day of 20 (E20), 23 (E23), 28 (E28), and 33 (E33). Our previous experiments showed that during this incubation window in ducks and also other poultry species a dramatic development of physiological mechanisms occurs. Further, during the last days of incubation the thermoregulatory system develops from an open loop system without feedback mechanisms into a closed feedback control system [1, 33, 36, 37]. During this period the duck embryos are more and more able to react on environmental influences nearly in an appropriate way. The eggs were incubated at 37.5°C and relative humidity of 60% (setter) and 80% (hatcher, from E28 until hatching). These conditions are common in artificial duck incubation. On the day of the experiment, three groups were formed. One group was incubated until the experiments at 37.5°C (control group). The second group was acute warm experienced at 39°C (warm group) and the third was acute cold experienced at 34°C (cold group) during 3 hours before the start of the experiments. It has to be noted that the stronger cold stimulation in comparison to the warm stimulation is necessary to induce changes in nNOS because of the high cold tolerance of bird embryos [40, 41].

The following experimental procedures were performed according to the European Community regulations and the German Low for Animal Protection.

### 2.2. nNOS Histochemistry

In the present study the nNOS activity was investigated in brain slices of the anterior hypothalamus from 80 Muscovy duck embryos using histochemistry.  Table 1 presents the number of embryos used for the experiments in each age group between E20 and E33.

Because nNOS and NADPH-d show similar biochemical features, in the present work the histochemical method of identification of the NADPH-d-containing neurons developed by Scherer-Singler and coworker [42] and modified by Hope and Vincent [43] was used.

The embryos were extracted from the eggshell and decapitated. Brains from the embryos were removed and fixed for a period of 90 minutes in 4% paraformaldehyde in phosphate buffer (0.1 M, pH 7.4) according to Matsumoto and co-worker [44]. The brains were washed six times for 30 minutes at 4°C in 0.1 M (pH 8.0) Tris-HCl solution (Tris-HCl). Later the brains were subjected to incubation in sucrose solutions of 10% and 25% in Tris-HCl for 1.5 and 12 hours, respectively.

Serial brain slices (25 *μ*m) were made using a cryostate (Leica Microsystems, Wetzlar). The slices containing the anterior hypothalamic region were attached to chromium gelatine-coated micro slides and washed in 0.1 M Tris-HCl for 5 min. Later they were incubated for 1-2 h in a solution containing NADPH-d (1 mM), nitro blue tetrazolium (0.5 mM), Triton X-100 (0.3%), and dicumarol (1 mM) in Tris-HCl at 22°C. Then slices were washed again in Tris-HCl solution for 5 minutes, dehydrated in ethanol, and covered with histokitt and glass coverslips. All chemicals used for the histochemistry were obtained from Sigma-Aldrich, Germany.

### 2.3. Data Analysis

**Figure 1:**
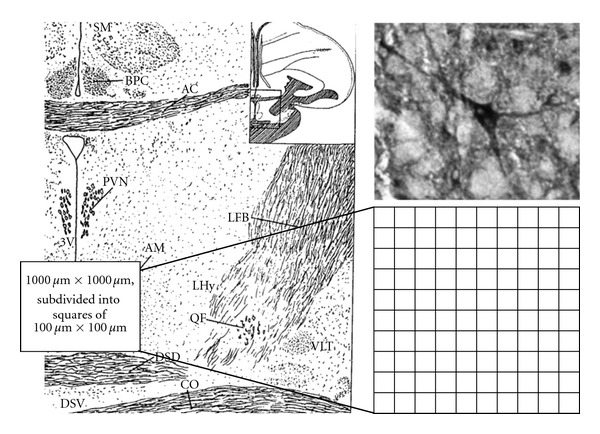
Left: anterior hypothalamus with the preoptic area after Kuenzel and Van Tienhoven [45], where the investigations were carried out (AC: *Commissura anterior*, PVN: *Nuc. paraventricularis*, 3V: third ventricle, AM: *Nuc. anterior medialis hypothalami, *CO: C*hiasma opticum*, LHy: lateral hypothalamic area, and FB: forebrain bundle). Right: typical examples of nNOS expression (magnification × 100) in a Muscovy duck embryo on E 23. nNOS positive Neurons were counted in a defined area, which was divided into 100 rectangles of 100 × 100 *μ*m.

For analysis, light microscopy and digital photography at a magnification of 40 fold (Zeiss Axioskop II, Zeiss AxioCam HRc) were used. NADPH-d (NOS)-positive neurons were counted in the anterior hypothalamus. Therefore, a rectangle mask (1000 *μ*m × 1000 *μ*m, subdivided into 100 squares of 100 *μ*m × 100 *μ*m) was placed near the third ventricle and the *nucleus anterior medialis hypothalami* above the optic chiasm (Figure 1). Stereotaxic data of the adult chicken brain were taken from Kuenzel and Masson [46] and proportionally adapted for the appliance in the embryonic duck brain. In relation to the age of the embryos, NOS-positive-neurons were counted in 4 to 8 brain slices, which contain the anterior hypothalamus with the preoptic area of the anterior hypothalamus (PO/AH).

Data were expressed as mean ± SD. The mean values were calculated over all of the 100 counted 100 *μ*m squares of the used rectangular mask in all brain slices (4–8), which were prepared from each duck embryo investigated (number of investigated duck embryos see Table 1)

Normal distribution of the data was tested using the Kolmogoroff-Smirnoff Test. The influence of temperature stimulation on nNOS expression in comparison to the control group was tested with Student's *t*-test for independent samples (if normal distributed) or using the Mann-Whitney *U*-test (if not normal distributed). The influence of embryonic age on nNOS expression was tested using the nonparametric Kruskal-Wallis test. Significance level was set at *P* < 0.05.

## 3. Results

**Figure 2:**
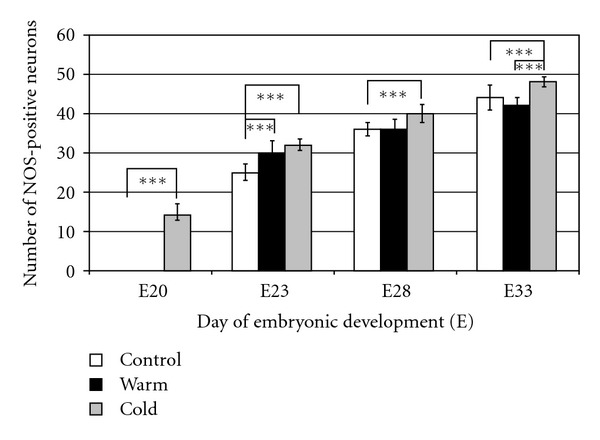
Hypothalamic nNOS expression in 20- to 33-days-old Muscovy duck embryos under different temperature treatments (control: constant 37.5°C, warm: 3 h 39°C, cold: 3 h 34°C before start of the experiments). Each column represents mean values ± SD of 100 (100 × 100 *μ*m) rectangle fields from 4–8 brain slices of each experimental group. Investigations were carried out in 80 embryos (see Table 1). Asterisks represent significance at the level of *P* < 0.001.

In all experimental series (control, cold, and warm stimulated group), a high significant (*P* < 0.001) increase in nNOS activity was found with increasing embryonic age. But on E20, in the control group as well as the warm stimulated group no nNOS-positive neurons were found; nNOS activity could be only observed in the cold stimulated group. Also in older embryos, the highest nNOS activity was obtained after 3 h cold stimulation in all age groups investigated (Figure 2). Between the control group and the cold stimulated group, the increase in nNOS activity was always of high significance (*P* < 0.001). A 3 h warm stimulation did not influence nNOS activity on E20 and E28. But a significant influence of warm stimulation on nNOS activity was found on E23 and E33. However, in comparison to the control group on E23 warm stimulation induced a statistically significant (*P* < 0.001) increase and on E33 a decrease (but not statistically significant) in the number of nNOS-positive neurons (Figure 2).

In summary, a clear influence on nNOS activity was shown after cold stimulation in all age groups of the duck embryos investigated, and always a high significant increase in nNOS was induced by the cold experience.

## 4. Discussion

In precocial birds, like ducks and chicken, the development of body functions starts early during embryogenesis. At the end of incubation, peripheral and central nervous thermoregulatory mechanisms, as well as other physiological functions, are well developed [36, 47]. Finally, the late-term embryo is able to react on changes in incubation temperature. If incubation temperature is increasing, Muscovy duck and chicken embryos react with increasing blood flow, activation of respiration and decrease in heat production [37, 48, 49]. Even under cold load, late-term poultry embryos are not able to keep body temperature constant, endothermic reactions occur when incubation temperature and body temperature are decreasing [37, 50]. It results in non or very small decrease in oxygen consumption. Under moderate cold load in Muscovy duck embryos, such endothermic counterreactions were already found on E22 [48]. Besides peripheral thermoregulatory mechanisms, a profound development of the neuronal structures in the anterior hypothalamus of Muscovy duck embryos during the second half of incubation corresponds to the electrophysiological findings. In Muscovy duck embryos during extracellular single neuronal recordings, thermosensitive PO/AH neurons were found on E22 and E23 [38], which show characteristics similar to the hatching [51] and posthatching period [34], growth period (own experiments, unpublished) and adult birds [52] as well as in mammals [53]. Our hypothesis is that early environmental stimulation of body functions improves their maturation and reactivity to environmental variations (“training effect”) during the perinatal period [33]. In chicken, for instance, short-term temperature “training” during the last 4 days of incubation improved vitality of hatched chicken and the posthatching growth [54]. Further, prenatal temperature manipulations may have long-term effects on the posthatching development and adaptability of body functions. In poultry, moderate chronic increase or decrease in incubation temperature during the last days of embryonic development induced postnatal changes in peripheral (e.g., thermoregulatory heat production, preferred ambient temperature, body temperature) and central nervous (e.g., neuronal hypothalamic thermosensitivity) mechanisms of thermoregulation, which are related to warm or cold adaptation [35, 55]. For instance, in 1-day-old Muscovy ducklings, which were long-term cold-incubated (34.5°C, E28 up to hatching), the thermoregulatory heat production under 1 h cold load of 10°C was significantly higher than in the normal incubated control group. These birds were able to keep their body temperature constant. The present investigations reveal that short-term low temperature application stimulates the activity of nNOS in all age groups investigated. Further, in comparison with warm stimulated embryos and the normal incubated control group, in short-term cold stimulated embryos nNOS activity was already detectable in an earlier stage of development. Both, the activation of nNOS on an earlier stage of development and the increasing expression of nNOS particularly after cold load in all other age groups investigated lead to the hypothesis that probably in bird embryos NO acts as a mediator of the neuronal cold pathway in the anterior hypothalamus. In adult mammals, the lateral hypothalamic area via the forebrain bundle controls different autonomic effectors of cold defense or heat conservation, for instance skin blood flow [56, 57], muscle shivering [58] and nonshivering thermogenesis in coordination with regulation of food intake [59]. The importance of NOS in cold-induced thermogenesis was described in rats. In cold environment systemic NOS inhibition by L-NAME injection induced a prolonged fall in body temperature, obvious by prevention of metabolic cold defense [60]. In rat pups aged between days 1 and 6, L-NAME applications abolished the oxygen consumption response to cold and reduced the metabolic cold defense in 10- to 11-day-old pups [14]. In opposite, in mammals under heat load [30], exercise [61], or fever [62] NO manly activates heat loss mechanisms. But in birds also under these conditions the role of NO seems to be different from that observed in mammals. In 5-days-old chicks under thermoneutral and heat stress conditions intramuscular as well as intracerebroventricular injections of NOS inhibitor L-NAME decreased body temperature and thus inhibited heat stress-induced hyperthermia [16].

However, in bird embryos, peripheral NO probably acts as a signal indicating the necessity for rewarming of the embryos [63]. NO emission was found mainly via the chorioallantoic membrane in these studies made on 18 bird species. Ar and coworkers [63] speculated that NO emission from eggs may carry a “message” from the embryo to the incubating parents and *vice versa*. The results from our study, which show a significant lower nNOS activity after short-term warm-load in comparison with the cold experienced group as well as the control group on E33, support the hypothesis that nNOS activity is a central (and obviously also peripheral) cold signal.

It is interesting that, in our study, in younger embryos short-term warm stimulation induced different changes in hypothalamic nNOS activity. Compared with the control on E23, an increase in nNOS was observed, too, and on E28 a warm load induced no change in nNOS. Only on E33 short-term warm- and cold-load caused opposite changes in nNOS compared with the control group. These different effects of short-term warm load on nNOS activity may be related to the peculiarity of early development of body functions that acute changes in environmental conditions induce as a rule, first uncoordinated and immediately nonadaptive reactions. Later the uncoordinated (immediately) nonadaptive reactions change into coordinated (adaptive) reactions obviously with the development of feedback mechanisms of the respective system [1, 35, 36]. For instance, experiments at the end of incubation time in chicken embryos revealed first proximate non-adaptive and later adaptive reactions with respect to the influence of cooling and warming on blood flow in the vessels of chorioallantoic membrane. In chicken embryos, the blood flow increased or decreased while warming or cooling on E15 until E19 (proximate non-adaptive). After this period, the reaction became proximate adaptive; on E20 and E21, the blood flow in the chorioallantoic membrane increased during warming and decreased during cooling, as expected. Similar changes in the blood flow during cooling were also found in Muscovy duck embryos at the end of incubation. However, in Muscovy duck embryos under warming proximate non-adaptive reactions persisted until day 34 of incubation [36].

In conclusion, the results show that short-term prenatal cold-load may stimulate nNOS activity in the anterior hypothalamus of Muscovy duck embryos during the entire investigated developmental period between E20 and E33. Warm stimulation obviously induces the opposite effect, but it starts at later embryonic age (E33). In younger duck embryos warm stimulation causes proximate nonadaptive reactions, similar with results we obtained in another study with respect to the blood flow in the vessels of chorioallantoic membrane. It can be concluded that probably in late-term bird embryos NO acts as a mediator of the neuronal cold pathway in the anterior hypothalamus.

## Figures and Tables

**Table 1:** Number of embryos investigated in each age group (E20 to E33) under control conditions (37.5°C), acute cold stimulation (34°C for 3 hours before experiment), and acute warm stimulation (39°C for 3 hours before experiment).

Applied temperature	Age of the embryos (E) in days			
	E20	E23	E28	E33
37°C	6	6	6	6
39°C	6	6	6	6
34°C	12	8	6	6
